# Subtypes in clinical burnout patients enrolled in an employee rehabilitation program: differences in burnout profiles, depression, and recovery/resources-stress balance

**DOI:** 10.1186/s12888-018-1589-y

**Published:** 2018-01-17

**Authors:** Kathrin Bauernhofer, Daniela Bassa, Markus Canazei, Paulino Jiménez, Manuela Paechter, Ilona Papousek, Andreas Fink, Elisabeth M. Weiss

**Affiliations:** 1 Department of Psychology, University of Graz, Universitätsplatz 2/DG, 8010 Graz, Austria; 2 Department of Psychology, University of Innsbruck, Bruno-Sander-Haus Innrain 52f, 6020 Innsbruck, Austria

**Keywords:** Burnout, Burnout subtypes, Burnout profiles, Person-oriented approach, Cluster analysis, Depression, Stress-recovery

## Abstract

**Background:**

Burnout is generally perceived a unified disorder with homogeneous symptomatology across people (exhaustion, cynicism, and reduced professional efficacy). However, increasing evidence points to intra-individual patterns of burnout symptoms in non-clinical samples such as students, athletes, healthy, and burned-out employees. Different burnout subtypes might therefore exist. Yet, burnout subtypes based on burnout profiles have hardly been explored in clinical patients, and the samples investigated in previous studies were rather heterogeneous including patients with various physical, psychological, and social limitations, symptoms, and disabilities. Therefore, the aim of this study is to explore burnout subtypes based on burnout profiles in clinically diagnosed burnout patients enrolled in an employee rehabilitation program, and to investigate whether the subtypes differ in depression, recovery/resources-stress balance, and sociodemographic characteristics.

**Methods:**

One hundred three patients (66 women, 37 men) with a clinical burnout diagnosis, who were enrolled in a 5 week employee rehabilitation program in two specialized psychosomatic clinics in Austria, completed a series of questionnaires including the Maslach Burnout Inventory – General Survey (MBI-GS), the Beck Depression Inventory, and the Recovery-Stress-Questionnaire for Work. Cluster analyses with the three MBI-GS subscales as clustering variables were used to identify the burnout subtypes. Subsequent multivariate/univariate analysis of variance and Pearson chi-square tests were performed to investigate differences in depression, recovery/resources-stress balance, and sociodemographic characteristics.

**Results:**

Three different burnout subtypes were discovered: the *exhausted* subtype, the *exhausted/cynical* subtype, and the *burned-out* subtype. The *burned-out* subtype and the *exhausted/cynical* subtype showed both more severe depression symptoms and a worse recovery/resources-stress balance than the *exhausted* subtype. Furthermore, the *burned-out* subtype was more depressed than the *exhausted/cynical* subtype, but no difference was observed between these two subtypes with regard to perceived stress, recovery, and resources. Sociodemographic characteristics were not associated with the subtypes.

**Conclusions:**

The present study indicates that there are different subtypes in clinical burnout patients (*exhausted*, *exhausted/cynical*, and *burned-out*), which might represent patients at different developmental stages in the burnout cycle. Future studies need to replicate the current findings, investigate the stability of the symptom patterns, and examine the efficacy of rehabilitation interventions in different subtypes.

## Background

During the last decade, burnout has become a public health issue affecting between 4% and 7% of the working population [1]. Nevertheless, the diagnosis is not yet included in clinical classification systems such as the Diagnostic and Statistical Manual of Mental Disorders (DSM-5) [2] or the International Statistical Classification of Diseases and Related Health Problems (ICD-10) [3]. To date, there is also no consensus on the definition of burnout and its core symptoms [4–7], and the diagnosis overlaps tremendously in symptomatology with other diagnoses, especially chronic fatigue [8] and depression [9–11].

Despite some conceptual controversies [4–7, 12], the most widely accepted definition of burnout is the one by Maslach, Schaufeli, and Leiter [6], who defined it as a work-related stress syndrome characterized by three main symptoms: exhaustion, cynicism, and reduced professional efficacy. Exhaustion refers to feelings of depletion caused by various work demands; cynicism reflects a distant attitude towards work and the people one is working with; and reduced professional efficacy represents the negative self-evaluation that one is incompetent and no longer able to perform work tasks adequately. However, past research has shown that burnout manifests in different ways. A varying number of burnout profiles [13–15] has been observed across studies, and multifaceted longitudinal trajectories of the burnout symptoms [16–21] indicate different pathways into burnout. Different burnout subtypes might therefore exist.

The person-oriented approach to burnout [15, 22] is one possibility to investigate burnout subtypes. It identifies different groups in a population that are intraindividually homogenous, but interindividually heterogeneous in various aspects such as symptomatology, psychopathology, and job- and person-related factors. In past research, the person-oriented approach has been used to identify typical burnout profiles [15], which might represent people at different developmental stages in the burnout cycle or truly different burnout subtypes [13, 23–27].

The most frequent burnout profiles that emerged in these studies [13–15], predominantly from the analysis of the three dimensions of the Maslach Burnout Inventory (MBI) [28], were the *burned-out* profile and the *healthy or engaged* profile representing people with severe or low symptomatology in all three burnout dimensions. Another common symptom profile was the *exhausted/cynical* profile characterized by high exhaustion and cynicism, but simultaneously high professional efficacy. Typical incongruent scoring patterns included the *exhausted or overextended*, the *cynical or disengaged*, and the *reduced professional efficacy* profile representing people with rather mild symptomatology where only one symptom is pronounced. However, one has to keep in mind that most previous studies have been conducted in non-clinical samples, which consist of people who report symptoms of a burnout but have not been diagnosed by a psychiatrist or clinical psychologist and who are all still working [13–15]. Therefore, the so-called healthy worker effect [29] occurred in these studies, and not all of the prior discussed burnout profiles might genuinely represent clinical burnout subtypes who need or seek treatment in specialized psychosomatic clinics or rehabilitation programs.

To date, only one study [30] has investigated subtypes based on burnout profiles in working-aged adults whose working ability was reduced or threatened by disease, disability or some other disorders, and who were therefore enrolled in an employee rehabilitation program. This study has identified four of the above mentioned subtypes: two subtypes with severe symptoms, namely the *burned-out* profile and the *exhausted/cynical* profile, one subtype with milder symptoms, namely the *reduced professional efficacy* profile, and the *healthy* profile. However, some of these profiles might have occurred because Hätinen et al. [30] explored a heterogeneous patient sample suffering from various physical, psychological, and social limitations, symptoms, and disabilities.

For that reason, the present study aims to examine whether meaningful burnout subtypes may be found in a more homogeneous group of clinically diagnosed burnout patients treated in a specialized psychosomatic clinic if the three main burnout symptoms suggested by Maslach, Schaufeli and Leiter [6] are considered and explored within a person-oriented approach [15]. Additionally, we will investigate the relationship of these subtypes to depression since an intense discussion has been going on during the last years [9–12, 31–54] whether burnout can be seen as a distinct construct or rather a new label of an already known state.

A growing body of literature has examined the overlap between burnout (subtypes) and depression-level, both in the working population [9, 12, 23, 31, 32, 39–48, 50–52] and in clinical patients [30, 54], but findings are heterogeneous and inconsistent. Several studies in the working population reported strong correlations between burnout and depression [9–11, 31, 32], especially between exhaustion and depression [10, 31, 32], while in clinically diagnosed burnout patients, the strong exhaustion-depression overlap could not be replicated [55]. Low to moderate correlations between burnout and depression were also found by others in the working population [40, 41, 47], whereas Ahola et al. [45] reported that burnout and depression overlapped particularly in severe burnout. These heterogeneous findings may partly result from using different measures of burnout and depression [11, 37] with some measures reflecting a larger concept redundancy between both disorders (for a discussion on this topic, see Maslach & Leiter [37]). Furthermore, longitudinal studies have been used to study the complex relationship between burnout and depression, but yielded inconsistent results as well. Some authors reported reciprocal relations between burnout and depression [42–44], while others found a unidirectional relationship from burnout to depression [46–50] or vice versa [51–53] or no predictive relation [36]. Studies applying a person-oriented approach to explore the burnout-depression overlap [9, 31] detected that burnout and depression were not separable from each other. Moreover, both disorders developed longitudinally in tandem supporting the hypothesis that burnout and depression may be the same disorder. In clinical burnout patients, longitudinal studies are still sparse [55]. Besides, only a few studies have explored the burnout-depression overlap by applying a person-oriented approach in clinical burnout patients [54] or burnout rehabilitation clients [30, 56], respectively. Yet, the overlap between burnout symptoms and depression might differ between burnout subtypes. Boersma and Lindblom [23] found in the working population that subtypes experiencing high exhaustion were more depressed than other burnout subtypes, and van Dam [54] could distinguish a group with mild symptoms from a group with severe symptoms on several measures (burnout, depression, anxiety, and fatigue) in clinically diagnosed burnout patients. In contrast, Hätinen et al. [30] found in working-aged rehabilitation clients that all subtypes were equally depressed, but in this study, a mixed patient sample suffering from various physiological, psychological, and social limitations, symptoms, and disabilities was explored.

Inconsistencies in organizational risk factors have been found in burnout subtypes as well. According to the job demands-resources (JD-R) model [57], burnout develops when job demands (e.g., workload, time pressure, conflict) are high, while resources (e.g., autonomy, social support, positive relationship with supervisor) are limited. Resources are therefore no longer able to buffer the negative impact of high demands on stress reactions [58]. Job demands and resources have also been linked to specific burnout symptoms: exhaustion is caused by high workload and emotional demands, whereas cynicism, reduced professional efficacy, and disengagement have been associated with a lack of resources [59–61]. Previous studies on burnout subtypes in the working population [13, 23, 27] likewise found that workload was high in subtypes experiencing high exhaustion (*burned-out*, *exhausted/cynical*, and *exhausted*), but resources were only low in subtypes with severe burnout symptoms (*burned-out* and *exhausted/cynical*), particularly in the *burned-out* subtype [13]. Yet, in clinical rehabilitation patients, Hätinen et al. [30] did not find any differences in job stressors and resources between three different burnout subtypes (*burned-out*, *exhausted/cynical*, and *low professional efficacy*), although recovery was associated with a decrease in job demands and an increase in job resources [56].

Because most prior research was conducted within the scope of the JD-R model of burnout, recovery has received comparatively less attention in relevant research. The recovery/resources-stress-balance model [62–64] is similar to the JD-R model but focusses additionally on recovery as crucial aspect to prevent burnout. According to the model, burnout develops after prolonged periods of stress without sufficient recovery and resources. More precisely, the homeostatic balance between stress and recovery is impaired because resources that were depleted during phases of stress are not adequately restored in the recovery phase [63]. The role of recovery has, however, not been systematically explored in burnout subtypes, particularly not in clinical burnout patients enrolled in an employee rehabilitation program.

Therefore, the current study aims to explore different burnout subtypes in clinical burnout patients, who are enrolled in an employee rehabilitation program in a psychosomatic clinic, by performing cluster analysis with the three subscales of the Maslach Burnout Inventory – General Survey (MBI-GS) [28] as clustering variables. Furthermore, depression levels will be compared among the subtypes, based on the expectation that the strength of the obviously existing overlap between burnout symptoms and depression might differ between burnout subtypes. Finally, differences between the burnout subtypes in the recovery/resources-stress balance and sociodemographic characteristics will be explored.

## Methods

### Study design and participants

This study used a person-oriented approach [15] to explore different subtypes based on burnout profiles in clinical burnout patients. A total of 103 patients (64% women) with a clinical burnout diagnosis were recruited from two specialized psychosomatic clinics in Austria. The patients were between 23 and 58 years old (*M* = 44.82 years, *SD* = 8.08) and had various educational backgrounds: 31% had a university degree or the A-level (high education), while 69% completed primary education, an apprenticeship or some other type of education or vocational training without the A-level (lower education).

### Procedure

Burnout diagnosis was established by a team of psychiatrists and clinical psychologists. Since separate diagnostic codes for burnout are not yet included in clinical classification systems such as DSM-5 or ICD-10 [2, 3], the burnout diagnosis was based on the ICD-10 criteria of work-related neurasthenia, which has been proposed as the psychiatric equivalent of clinical burnout [38]. Similar to previous studies [54, 65], we included only patients that scored ≥2.20 on exhaustion and either ≥2.00 on cynicism or ≤3.67 on professional efficacy in the MBI-GS. Due to their burnout symptoms, all patients were enrolled in a 5 week employee rehabilitation program at the clinics, and they were on sick leave for at least 1 week prior to study admission (*M* = 106.20 days, *SD* = 113.46). Data were collected during the first week of the rehabilitation program and informed consent was obtained from all patients prior to participation. The study was in accordance with the 1964 Declaration of Helsinki and was approved by the ethics committee of the University of Graz, Austria.

### Psychometric measures

#### Burnout

Burnout symptom severity was assessed with the German version [66] of the MBI-GS [28]. It has 16 items and consists of three subscales: exhaustion, cynicism, and reduced professional efficacy. The exhaustion subscale consists of five items (e.g., feeling emotionally drained from work), the cynicism subscale includes also five items (e.g., enthusiasm has decreased since work was started), and the professional efficacy subscale consists of six items (e.g., feeling that one gets done things effectively). The items are answered on a frequency rating scale ranging from 0 (never) to 6 (daily). Since all participants were on sick leave, they were instructed to respond to the items of the MBI-GS according to how they would feel if they were working at the moment.

#### Depression

The German version of the Beck Depression Inventory (BDI) was used to assess depression severity [67]. The BDI is a 21-item self-rating questionnaire that covers a variety of depressive symptoms along a continuum from 0 (absent or mild) to 3 (severe) symptoms. The BDI is widely used in treatment settings. A cut-off score ≥ 18 indicates a clinically relevant level of depression [68]. Additionally, all patients were screened for a past diagnosis of major depression using the Structured Clinical Interview for Axis I DSM-IV Disorders (SCID-I) [69].

#### Recovery/resources-stress balance

The Recovery-Stress-Questionnaire for Work (RESTQ-Work) is based on the recovery/resources-stress balance model [63] and consists overall of 92 items [62]. On a 7-point frequency rating scale ranging from 0 (never) to 6 (always), it measures the degree of stress and the extent of recovery and resources in the past 7 days/nights. The RESTQ-Work has seven subscales: *social-emotional stress* (e.g., being mentally stressed, irritated, and frequently in arguments with others), *performance (−related) stress* (e.g., time pressure, interruptions at work), *loss of meaning/burnout* (e.g., emotional exhaustion, loss of control, meaninglessness), *overall recovery* (e.g., physical recovery, relaxation, satisfying sleep), *leisure/breaks* (e.g., undisturbed leisure time without too many high-duty activities such as household chores, efficient breaks at work), *psychosocial resources* (e.g., social support from family, friends, and colleagues), and *work-related resources* (e.g., autonomy, participation, experience of personal growth). The RESTQ-Work displays good internal reliability and validity, and it has been used in burnout research before [58].

### Statistical analyses

The burnout-depression overlap for the entire patient sample was analyzed using basic correlations and correlations corrected for attenuation [70] between the three MBI-GS subscales and the BDI score. To explore different burnout subtypes, cluster analyses [71] were performed. The three MBI-GS subscales (exhaustion, cynicism, and professional efficacy) served as the clustering variables and were standardized (z-score; *M* = 0, *SD* = 1) prior to the analyses. First, a hierarchical agglomerative cluster analysis with Ward’s method as a linkage method and squared Euclidean distance as a similarity measure was conducted. The optimal number of clusters was determined by the dendrogram, which is a visual illustration of how the individual cases are arranged into the clusters produced by hierarchical clustering. Next, a K-means cluster analysis was conducted to improve the cluster fit [71]. To identify burnout subtypes based on burnout profiles, the same approach has been applied by others as well [23, 30].

To describe the burnout subtypes further, one-way analyses of variance (ANOVAs) with the clusters as independent variable and the three MBI-GS subscales as dependent variables were conducted. Differences in depression between the subtypes were evaluated using Pearson’s chi-square tests (for BDI ≥ 18 and depression diagnosis in the past) and a one-way ANOVA. Differences between the subtypes in recovery/resources-stress balance were evaluated using a one-way MANOVA. In the ANOVAs and MANOVA, the clusters served as the independent variable, while the BDI and the seven RESTQ-Work subscales were the dependent variables, respectively. Sociodemographic differences between the subtypes were investigated with one-way ANOVAs (for age and days on sick leave) and Pearson’s chi-square tests (for gender, education, and rehabilitation clinic). In case of homogeneous variances, post-hoc comparisons were made with the Bonferroni test; in case of inhomogeneous variances, the Games-Howell test was used. Effect sizes are reported as partial eta-square (η_p_^2^) indicating small (0.01 ≤ η_p_^2^ < 0.06), medium (0.06 ≤ η_p_^2^ < 0.14), and large (η_p_^2^ ≥ 0.14) effects, respectively [72]. All statistical procedures were calculated in SPSS 24 and were performed with α = 0.05 (two-tailed).

## Results

### Burnout-depression overlap in the entire patient sample

To explore the burnout-depression overlap in the entire patient sample, basic correlations and correlations corrected for attenuation [70] between the MBI-GS subscales and the BDI were inspected (see Table 1). The three burnout subscales correlated moderately with depression. The highest correlation emerged between cynicism and depression (*r* = 0.41; *p* < 0.01), the lowest correlation occurred between exhaustion and depression (*r* = 0.30; *p* < 0.01). Regarding the correlations between the three MBI-GS subscales, both exhaustion and cynicism (*r* = 0.53; *p* < 0.01) and cynicism and professional efficacy (*r* = −0.51; *p* < 0.01) correlated strongly with each other, whereas a low correlation emerged between exhaustion and professional efficacy (*r* = −0.16; *p* = 0.10).

**Table 1:** Cronbach’s alpha (α) and correlations (correlations corrected for attenuation are in italics) between the MBI-GS subscales and the BDI

	1.	2.	3.	4.	α
1. MBI – exhaustion	1	0.53**	−0.16	0.30**	0.86
2. MBI – cynicism	0.65**	1	−0.51**	0.41**	0.77
3. MBI – professional efficacy	−0.20*	−0.66**	1	−0.35**	0.78
4. BDI	0.35**	0.51**	−0.42**	1	0.88

*p < 0.05; **p < 0.01

### Burnout subtypes in clinically diagnosed burnout patients

Cluster analysis with the three MBI-GS subscales indicated two to four burnout subtypes in the present sample. In the two-cluster solution, cluster 1 and cluster 2 grouped together although the patients of cluster 2 had more severe burnout symptoms than the patients of cluster 1 (see Table 2). In the four-cluster solution, a cluster of patients with elevated burnout symptoms emerged that lay in between cluster 1 and cluster 2. However, since the four-cluster solution did not provide more conceptual clarity than the three-cluster solution, we considered the three-cluster solution representing three different burnout subtypes the best. Subsequent ANOVAs showed how the three subtypes differed in burnout symptomatology (see Table 2).

**Table 2:** Differences between the burnout subtypes in burnout, depression, and recovery/resources-stress balance

			subtype 1		subtype 2		subtype 3				
	overall (*N* = 103)		exhausted (*N* = 34)		exhausted/ cynical (*N* = 39)		burned-out (*N* = 30)				
	*M*	*SD*	*M*	*SD*	*M*	*SD*	*M*	*SD*	*F*(2,100)	η_p_^2^	contrasts
MBI											
exhaustion	5.06	0.84	4.13	0.62	5.60	0.37	5.43	0.54	84.51**	0.63	3,2 > 1
cynicism	4.13	1.01	3.28	0.71	4.17	0.85	5.03	0.63	43.84**	0.47	3 > 2 > 1
professional efficacy	4.56	0.80	4.83	0.55	5.05	0.43	3.60	0.54	77.73**	0.61	1,2 > 3
BDI											
depression	21.46	9.65	16.41	8.68	21.31	8.74	27.39	8.69	12.70**	0.20	3 > 2 > 1
RESTQ-Work											
social-emotional stress	2.58	1.12	1.88	0.79	2.69	1.18	3.22	0.95	14.85**	0.23	3,2 > 1
performance (−related) stress	3.08	1.10	2.32	0.92	3.32	0.94	3.63	1.03	16.96**	0.25	3,2 > 1
loss of meaning/burnout	3.04	1.20	2.04	0.83	3.47	0.94	3.62	1.18	26.63**	0.35	3,2 > 1
overall recovery	2.12	0.81	2.65	0.77	1.98	0.74	1.70	0.61	15.27**	0.23	1 > 2,3
leisure/breaks	2.95	1.21	3.56	1.13	2.72	1.12	2.56	1.18	7.39**	0.13	1 > 2,3
psychosocial resources	2.88	1.28	3.34	0.98	2.75	1.37	2.51	1.34	3.84*	0.07	1 > 3
work-related resources	2.70	0.90	3.02	0.86	2.73	0.86	2.31	0.85	5.39**	0.10	1 > 3

*p < 0.05; **p < 0.01

**Fig. 1:**
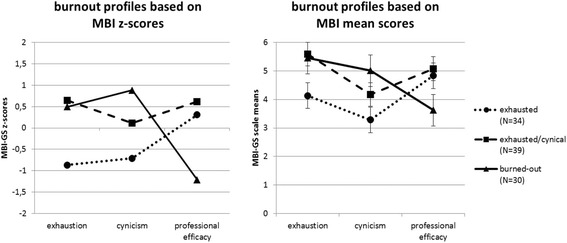
Burnout profiles based on MBI z-scores and based on MBI mean scores. ^a^ subtype 1 = *exhausted*; subtype 2 = *exhausted/cynical*; subtype 3 = *burned-out*. ^b^ post-hoc tests MBI-GS: exhaustion (*burned-out subtype* vs. *exhausted subtype*, *p* < 0.01; *exhausted/cynical* subtype vs. *exhausted* subtype, *p* < 0.01; *burned-out* subtype vs. *exhausted/cynical* subtype, *p* = 0.30); cynicism (*burned-out subtype* vs. *exhausted subtype*, *p* < 0.01; *exhausted/cynical* subtype vs. *exhausted* subtype, *p* < 0.01; *burned-out* subtype vs. *exhausted/cynical*, *p* < 0.01); professional efficacy (*burned-out subtype* vs. *exhausted subtype*, *p* < 0.01; *exhausted/cynical* subtype vs. *exhausted* subtype, *p* = 0.20; *burned-out* subtype vs. *exhausted/cynical*, *p* < 0.01).

Figure 1 displays the symptom profile of the burnout subtypes graphically, both in z-scores and in mean scores. Each burnout subtype was described based on its mean score profile and based on which symptoms were most pronounced within the subtype. The three subtypes were labeled as follows: *burned-out* (subtype 3), *exhausted/cynical* (subtype 2)*,* and *exhausted* (subtype 1). The *burned-out* subtype (*n* = 30) had the most severe burnout symptomatology. It displayed high exhaustion, high cynicism, and low professional efficacy. The *exhausted/cynical* subtype (*n* = 39) had severe burnout symptoms as well. It was characterized by high exhaustion, elevated levels of cynicism, but simultaneously high professional efficacy. Finally, the *exhausted* subtype (*n* = 34) showed the least severe burnout symptoms. It displayed elevated levels of exhaustion, the lowest scores on cynicism, and high professional efficacy.

Based on the z-score profile, the three subtypes could have been labeled as *burned-out* (subtype 3), *exhausted* (subtype 2), and *healthy* (subtype 1). However, we decided to label the subtypes based on the means score profile because of the general higher symptom severity in clinical burnout patients. Based on the z-score profile, particularly the label *healthy* for subtype 1 and the label *exhausted* for subtype 2 would have been misleading because both subtypes had elevated levels of exhaustion and subtype 2 had additionally elevated levels of cynicism. Hence, subtype 1 did not seem to be healthy and subtype 2 did not seem to be solely exhausted.

### Level of depression in the burnout subtypes

Depression score was highest in the *burned-out* subtype, followed by the *exhausted/cynical* subtype, and the *exhausted* subtype (see Table 2; Bonferroni-corrected post-hoc tests: *burned-out* subtype vs. *exhausted/cynical* subtype, *p* = 0.02; *burned-out* subtype vs. *exhausted* subtype, *p* < 0.01; *exhausted/cynical* subtype vs. *exhausted* subtype, *p* = 0.05). Moreover, there were significant differences between the subtypes with regard to how many patients scored above the BDI cut-off score ≥ 18 for clinical depression (*burned-out* subtype: 27 patients (90%); *exhausted/cynical* subtype: 27 patients (69%); *exhausted* subtype: 14 patients (41%); *χ*^2^ (2, *N* = 103) = 17.22, *p* < 0.01). Concerning previous depression diagnosis, no significant difference was observed between the subtypes, *χ*^2^ (2, *N* = 103) = 0.77, *p* = 0.68, (see Table 3).

**Table 3:** Sociodemographic characteristics of the burnout subtypes

			subtype 1	subtype 2	subtype 3
		overall (*N* = 103)	exhausted (*N* = 34)	exhausted/ cynical (*N* = 39)	burned-out (*N* = 30)
gender	female	66 (64%)	21 (32%)	24 (36%)	21 (32%)
	male	37 (36%)	13 (35%)	15 (41%)	09 (24%)
education	high	32 (31%)	08 (25%)	12 (38%)	12 (38%)
	lower	71 (69%)	26 (37%)	27 (38%)	18 (25%)
BDI ≥ 18	yes	68 (66%)	14 (21%)	27 (40%)	27 (40%)
	no	35 (34%)	20 (57%)	12 (34%)	03 (9%)
depression diagnosis in the past	yes	70 (68%)	25 (36%)	25 (36%)	20 (29%)
	no	33 (32%)	09 (27%)	14 (42%)	10 (30%)
rehabilitation clinic^a^	A	51 (49%)	15 (29%)	21 (41%)	15 (29%)
	B	52 (51%)	19 (37%)	18 (35%)	15 (29%)
age	*M* (*SD*)	44.82 (8.08)	44.96 (9.72)	45.76 (6.26)	43.43 (8.22)
days sick leave	*M* (*SD*)	106.20 (113.46)	73.65 (94.66)	115.56 (104.04)	130.93 (137.13)

^a^data from two different rehabilitation clinics

### Level of recovery/resources-stress balance in the burnout subtypes

The MANOVA showed significant differences in recovery/resources-stress balance between the three subtypes (using Pillai’s trace, *V* = 0.48, *F*(14, 190) = 4.33, *p* < 0.01, η_p_^2^ = 0.24). Subsequent ANOVAs indicated significant differences in all seven RESTQ-Work subscales (see Table 2). Post-hoc analyses further revealed that both the *burned-out* and the *exhausted/cynical* subtype experienced significantly more social-emotional stress (*p* < 0.01), performance(−related) stress (*p* < 0.01), and loss of meaning/burnout (*p* < 0.01) as well as significantly less overall recovery (*p* < 0.01) and less leisure/breaks during work (*p* < 0.01) than the *exhausted* subtype. Furthermore, the *burned-out* subtype had significantly less psychosocial resources (*p* = 0.02) and less work-related resources (*p* < 0.01) than the *exhausted* subtype, while the *burned-out* and the *exhausted/cynical* subtype did not differ from each other in any of the RESTQ-Work subscales.

### Sociodemographic characteristics of the burnout subtypes

Sociodemographic data for the burnout subtypes are presented in Table 3. There were no significant differences between the three subtypes with regard to sociodemographic characteristics. Men and women were equally distributed across the three clusters, *χ*^2^ (2, *N* = 103) = 0.65, *p* = 0.72, and the subtypes did not differ in age, *F*(2, 100) = 0.71, *p* = 0.50, and education, *χ*^2^ (2, *N* = 103) = 2.02, *p* = 0.36, respectively. The rehabilitation clinic was not significantly associated with the burnout subtype, *χ*^2^ (2, *N* = 103) = 0.69, *p* = 0.71, and there was no significant difference between the subtypes in how many days the patients had already been on sick leave prior to study admission, *F*(2, 100) = 2.30, *p* = 0.10.

## Discussion

### Burnout subtypes in clinical burnout patients

The present study explored different burnout subtypes based on MBI profiles in clinical burnout patients enrolled in an employee rehabilitation program in a psychosomatic clinic. As increasing evidence points to intra-individual patterns of burnout symptoms in non-clinical samples such as students, athletes, healthy and burned-out employees [13, 15], this study addressed the specific research question of whether different burnout subtypes can also be identified in clinically diagnosed burnout patients and whether these burnout subtypes differ in depression, recovery/resources-stress balance, and sociodemographic characteristics. Previously, van Dam [54] investigated subgroups in clinically diagnosed burnout patients by means of cluster analysis using fatigue (CIS), depression (SCL-90-D), and anxiety (SCL-90-A) as clustering variables and found two clusters that differed from one another in terms of symptom-severity on the three aforementioned measures. To date, only Hätinen et al. [30] have explored MBI burnout profiles in a mixed sample of working-aged rehabilitation clients, in which four different symptom patterns occurred: the *burned-out* profile, the *exhausted/cynical* profile, the *reduced professional efficacy* profile, and the *healthy* profile. However, some of these profiles might have occurred because Hätinen et al. [30] explored a heterogeneous patient sample suffering from various physical, psychological, and social limitations, symptoms, and disabilities. A study analyzing burnout subtypes in a group of clinically diagnosed burnout patients with the three MBI-GS dimensions (exhaustion, cynicism, professional efficacy) as clustering variables, is to the very best of our knowledge not available yet. Cluster analysis with the three MBI-GS subscales as clustering variables revealed three distinct burnout subtypes in the present study.

In line with Hätinen et al. [30], we found two subtypes with severe burnout symptoms, namely the *burned-out* and the *exhausted/cynical* subtype, whereas the *reduced professional efficacy* subtype and the healthy burnout profile could not be replicated in the present study. Instead, we identified an *exhausted* subtype representing patients with milder symptomatology. Sociodemographic characteristics were not systematically associated with the subtypes in the present study, but all groups differed in depression severity. The *burned-out* subtype showed overall the highest level of depression, while the *exhausted* subtype was the least depressed. Moreover, the *burned-out* and the *exhausted/cynical* subtype had a worse recovery/resources-stress balance compared to the *exhausted* subtype, but they did not differ from each in perceived stress, recovery, and resources. Since neither the depression scale, nor the RESTQ-Work subscales were used for clustering, these differences further support the validity of the cluster solution. Taken together our findings suggest that different burnout subtypes can be identified in clinical patients who were diagnosed with burnout by a professional team of psychiatrists and clinical psychologists working in a specialized psychosomatic clinic.

In line with past research [30, 54], our study indicates that the three subtypes represent patients at different stages in the burnout cycle. The subtypes correspond well with the process model of burnout [18], according to which burnout starts with exhaustion due to prolonged periods of stress, followed by cynicism as an attempt to cope with the intense emotional strain, before finally reduced professional efficacy occurs because a negative attitude towards one’s job precludes achieving work goals. Yet, it cannot be ruled out that the *burned-out* and the *exhausted/cynical* subtype might be truly different burnout subtypes, each characterizing the final stage in the burnout cycle. Several researchers have shown that professional efficacy develops rather independently of the other two burnout symptoms [21, 60], and both burnout profiles seem to be relatively stable over time [23]. Moreover, an alternative longitudinal trajectory of the burnout symptoms starting with reduced professional efficacy, followed by cynicism, and ending in emotional exhaustion, has been found as well [21]. The *reduced professional efficacy* subtype is a common subtype in the general working population [15], and two different pathways into burnout—one starting with exhaustion, the other one starting with reduced professional efficacy—have been addressed [13, 15, 24].

Truly different burnout subtypes might also explain why Hätinen et al. [30] found the *reduced professional efficacy* subtype, while the present study found the *exhausted* subtype representing patients with milder symptomatology, although the same methodology was applied in both studies, namely cluster analysis with the three MBI-GS subscales. Nevertheless, one has to keep in mind that our study sample markedly differed from the rehabilitation clients analyzed in the study by Hätinen et al. [30]. The present study solely included clinical burnout patients diagnosed with the ICD-10 criteria of work-related neurasthenia, which has been proposed as the psychiatric equivalent of clinical burnout [38]. In addition, all patients scored high on exhaustion (≥ 2.20) and either high on cynicism (≥ 2.00) or low on professional efficacy (≤ 3.67) in the MBI-GS [54, 65]. In contrast, Hätinen et al. [30] explored a mixed patient sample from an employee rehabilitation clinic suffering from various physical, psychological, and social limitations, symptoms, and disabilities. Patients with physical impairments displayed particularly the *healthy* burnout profile in their study, but physical impairments can lead to the experience of reduced professional efficacy as well, when one is no longer able to conduct one’s job properly.

Regarding the possible antecedents of burnout, we revealed that the three burnout subtypes in our study differed in their recovery/resources-stress balance. The two subtypes with severe burnout symptoms, namely the *burned-out* subtype and the *exhausted/cynical* subtype, experienced more social-emotional stress, performance(−related) stress, and loss of meaning in their job than the *exhausted* subtype. Both subtypes reported also the lowest overall recovery, leisure, and breaks during work. Furthermore, the *burned-out* subtype tended to have less psychosocial and work-related resources than the *exhausted* subtype. According to the JD-R model [57] and the recovery/resources-stress balance model [62–64], burnout develops due to high job demands and simultaneously low resources and recovery. Both would be necessary to buffer the negative effects of high job demands on stress reactions [58]. Our findings are in line with these assumptions, which have also received support in burnout subtypes in the working population [13, 23, 27] and in longitudinal studies in clinical rehabilitation clients [56]. Yet, the *burned-out* and the *exhausted/cynical* subtype did not differ in recovery/resources-stress balance from each other in the present study. This is in line with some previous studies [23, 30] but not with others [13] reporting that the *burned-out* subtype had even less organizational resources than the *exhausted/cynical* subtype. To clarify, whether the *exhausted/cynical* subtype and the *burned-out* subtype represent people at different developmental stages in the burnout cycle or truly different burnout subtypes, future studies should explore longitudinally which characteristics differentiate between these two subtypes.

### Burnout-depression overlap

During the last years, an intense discussion has been going on whether burnout can be seen as a distinct construct or rather a new label of an already known state, and much of this debate has focused around the burnout-depression overlap [9–12, 31–54]. Until now, a vast majority of studies confirms that burnout and depression are not independent and especially in severe burnout, both disorders strongly overlap [11, 32, 45, 54]. This was also demonstrated in our own data. In the present study, 90% of the patients in the *burned-out* subtype scored above the BDI cut-off score ≥ 18 for clinical depression [68], whereas only 69% / 41% of the patients reached this threshold in the *exhausted/cynical* subtype and the *exhausted* subtype, respectively. Similarly, Ahola et al. [45] showed that 90% of people with severe burnout reported a physical or mental disease; more specifically, pain and depression. In contrast, Hätinen et al. [30] could not replicate these findings; the authors found that several burnout subtypes in rehabilitation clients were equally depressed. Yet, in their study, a mixed sample of rehabilitation clients suffering from various physiological, psychological, and social limitations, symptoms, and disorders was explored, while other studies [23, 54] using a person-oriented approach to explore burnout subtypes found that subtypes with severe burnout symptoms had higher depression scores than subtypes with milder burnout symptoms, which is in line with our study.

Interestingly, our results differed also from previous findings with regard to the burnout-depression overlap when the correlations between the three MBI-GS subscales and the BDI score were considered [9, 10, 31, 32]. For example, Bianchi et al. [10] found a high correlation between the MBI sum score and the BDI (*r* = 0.68) in the working population. Importantly, in their study, the correlation between emotional exhaustion and depression was much stronger (*r* = 0.74) than the correlations between the three dimensions of the MBI. Similar to the study of van Dam [54], the present study could not replicate such a strong correlation between exhaustion and depression (*r* = 0.30) in clinical burnout patients. For more than half a century [73], researchers have debated about the utility of using non-clinical samples such as students as analogues for patients with clinical diagnosis. Generalizing findings from non-clinical burnout samples to clinical burnout patients might therefore be problematic. However, another explanation for the lower correlation between exhaustion and depression in the present study and in the study of van Dam [54] might be that the range of exhaustion and depression scores is limited in clinical burnout patients, which might have flattened the correlation between both constructs. Furthermore, some of the heterogeneity in study results concerning the burnout-depression overlap may result from using different measures of burnout and depression [11, 12, 31, 32, 37, 40, 41, 47]. Hence, the high correlations between burnout and depression, especially with the burnout dimension emotional exhaustion in some studies, might partly reflect a large level of concept redundancy between specific burnout and depression measures (for a discussion on this topic, please see Maslach & Leiter [37]).

Especially longitudinal studies provide the opportunity to study the complex relationship between burnout and depression. Longitudinal studies focusing on the question whether burnout predicted depression or vice versa, revealed inconsistent results. Some authors [42–44] found a reciprocal relation between burnout and depression, with each predicting subsequent developments in the other, while other authors [46–50] reported a predictive relation from burnout to depression. Furthermore, three longitudinal studies showed a unidirectional relationship from depression to burnout [51–53] and one study failed to find any predictive relation [36]. Bianchi, Schonfeld and Laurent [31] focused on how burnout and depressive symptoms clustered at baseline and follow-up by applying a person-oriented approach. In this study, burnout was measured with a global burnout index (the combination of the MBI subscales emotional exhaustion and cynicism); depression was assessed with the Patient Health Questionnaire (PHQ-9). A similar person-oriented approach by using cluster analysis to study the relationship between burnout and depressive symptoms at baseline and over 7 years in dentists was applied by Ahola et al. [9]. Again, burnout was measured with a global index (more specifically, a weighted sum score of the MBI dimensions was calculated, so that emotional exhaustion, depersonalization, and diminished personal accomplishment had different weights in the syndrome); depressive symptoms were assessed using the short form of the Beck Depression Inventory (BDI-SF). In both studies, similar findings were obtained as both studies could show that the participants formed three clusters with low, intermediate, or high levels of burnout and depressive symptoms at baseline. Additionally, in both studies long-term development occurred in tandem, either remaining stable or changing in synchrony. Therefore, both studies support the hypothesis that burnout and depression overlap or may even be the same disorder.

Up to now, longitudinal studies in clinical burnout patients are still sparse. Oosterholt et al. [55] examined the course of cognitive performance and cortisol level in clinical burnout patients and non-clinical burnout individuals over a time period of 1.5 years. After 1.5 years, clinical burnout patients showed improvement of burnout symptoms and general physical and psychological complaints, but the patients still reported subjective cognitive impairments that were also evident in a cognitive test. In contrast, the non-clinical burnout group expressed the same elevated level of burnout symptoms, general physical and psychological complaints, and cognitive problems, but did not show objective cognitive deficits after 1.5 years.

Hätinen et al. [30] used a person-oriented approach to explore MBI burnout profiles and the changes in burnout 4 months after a rehabilitation intervention in a mixed sample of working-aged rehabilitation clients. In this study, four different burnout profiles (*burned-out* profile, *exhausted/cynical* profile, *reduced professional efficacy* profile, and *healthy* profile) were found and the burnout changes observed 4-month after the intervention depended on the burnout profile membership. While exhaustion showed a decreasing trend in the *burned-out* and *exhausted/cynical* profile, the decreasing trend in cynicism was only evident in the *burned-out* profile compared to the other profiles. Furthermore, reduced professional efficacy showed an increasing trend in the *exhausted/cynical* profile, whereas in the *reduced professional efficacy* profile the trend was decreasing. In a subsequent study, Hätinen et al. [56] investigated burnout trajectories in a one-year rehabilitation program with a six-month follow-up and found inter-individual changes in how clients reacted to the interventions and how these reactions were manifested in burnout symptoms during follow-up. The authors found three burnout patterns: two patterns with high levels of burnout and one pattern with low levels of burnout. However, only in one group with high burnout levels, exhaustion and cynicism were decreased at follow-up. This group was labeled as “high burnout-benefited trajectory” by the authors. For the “low burnout trajectory” and the “high burnout-benefited trajectory”, positive changes were detected in job-related antecedents (e.g., time pressure at work, job control, workplace climate) and consequences (e.g. depressive symptoms, job satisfaction). Thus, preliminary results suggest that in clinical burnout samples distinct symptom profiles exist and that not all burnout profiles benefit equally from rehabilitation in terms of reduction in burnout symptoms, job-related antecedents, and consequences [30, 56].

### Practical implications

Burnout prevention/intervention programs in non-clinical burnout employees are either person-directed (individual level interventions), organization-directed or a combination of both [74]. Person-directed interventions usually consist of different kinds of relaxation exercises and/or cognitive behavioral techniques to enhance social support, job competence, and personal coping skills. Organization-directed interventions target on poor workplace climate, dissatisfaction with supervision, and changes in work procedures such as task restructuring. Furthermore, organization-directed interventions aim at decreasing job demand, increasing job control and expanding the level of participation in decision-making. Several reviews in non-clinical populations [74–77] concluded that burnout intervention programs had a beneficial effect on burnout outcome measures, especially when person-directed and organization-directed interventions were combined. Nonetheless, a recent meta-analysis [78] on the effectiveness of controlled interventions to reduce burnout indicated statistically significant effects of interventions only on general burnout scales (*d* = 0.224) and exhaustion (*d* = 0.172); no effect was observed on cynicism or professional inefficacy. Moreover, the reported effect sizes were overall rather small. Yet, most studies included in this meta-analysis investigated non-clinical burnout employees who do not necessarily possess a high level of burnout, since the interventions were aimed at decreasing the level of stress.

Studies assessing the efficacy of interventions in burnout patients who are treated in clinical settings are rare [30, 55, 56]. Preliminary results suggest that in clinical burnout samples distinct symptom profiles exist and that not all burnout profiles benefit equally from rehabilitation in terms of reduction in burnout symptoms, job-related antecedents, and consequences [30, 56]. For the burnout subtypes with severe symptomatology such as the *exhausted/cynical* and the *burned-out* subtype, the first step in rehabilitation should lie on the alleviation of the burnout and depression symptoms (e.g., psychotherapy and if indicated antidepressants). Additionally, rehabilitation should focus on organization-directed intervention activities in these subtypes, since burnout patients with severe symptomatology reported worse interpersonal relations, poor workplace climate, and dissatisfaction with supervision in previous studies [30]. Similarly, in our own study, the *burned-out* and the *exhausted/cynical* subtype experienced high social-emotional stress, performance-related stress and limited (work-related) resources. There is still an ongoing debate about the efficacy of antidepressants and psychotherapies in the treatment of subclinical depression, which is defined as a level of clinically relevant depressive symptoms in the absence of a major depressive disorder [79, 80]. Therefore, the *exhausted* subtype might profit from a person-directed approach focusing on factors contributing to exhaustion and recovery (e.g., interventions targeting workload, relaxation, and work-life balance [27]. In sum, identifying different subtypes of clinical burnout patients and analyzing the relationship of these subtypes to depression will enable psychiatrists and clinical psychologists to focus their intervention activities more effectively.

### Study limitations

Several limitations of the current study need to be addressed. Similar to previous studies [54, 65], in the current paper the term clinical burnout is used to distinguish our study population of clinically diagnosed patients treated in a specialized psychosomatic clinic from non-clinical burnout groups who report symptoms of a burnout but are neither diagnosed by a psychiatrist or clinical psychologist as such and who are all still working. To date, there are no consensual diagnostic criteria for the burnout syndrome and the disorder is not yet included in clinical classification systems such as DSM-5 or ICD-10 [2, 3]. Therefore, in the current study, the burnout diagnosis was based on the ICD-10 criteria of work-related neurasthenia, which has been proposed as the psychiatric equivalent of clinical burnout [38]. Additionally, we included only patients who scored ≥2.20 on exhaustion and either ≥2.00 on cynicism or ≤3.67 on professional efficacy in the MBI-GS. Hence, the findings of our study might not be generalizable to clinical burnout patients who are diagnosed differently. An extensive discussion about the problem of simplification burnout to ICD-10/DSM-5 codes such as neurasthenia, somatic symptom disorder or exhaustion, can be found in a current review by Maslach and Leiter [37].

Furthermore, the patients in the present study were on average three and a half months on sick leave, and hence, a recall bias may have occurred; especially in patients who had been longer on sick leave. However, the burnout subtypes did not differ in how many days the patients had been on sick leave; thus, the distortion bias was at least equally distributed between the subtypes. Additionally, data were collected during the first week of the rehabilitation program. Therefore, it cannot be ruled out that the burnout profiles we found occurred because some patients already started benefiting from the positive effects of the rehabilitation program before they filled in the questionnaires. A further limitation of the present study is that the patients were not screened for comorbid psychiatric disorders using the Structured Clinical Interview for Axis I DSM-IV Disorders (SCID-I) [69] or the Mini-International Neuropsychiatric Interview (MINI) [81]. Therefore, we cannot ensure that comorbid disorder are equally divided over the burnout types. Finally, the analyses were based on cross-sectional report questionnaires. Hence, we were not able to investigate whether the *exhausted/cynical* and the *burned-out* subtype represent people at different developmental stages in the burnout cycle or truly different burnout subtypes. Due to the rather small sample size and different interventions in the two rehabilitation clinics, we were also not able to investigate if the three burnout subtypes responded differently to therapy.

## Conclusions

In recent years, different burnout subtypes or profiles, based on cluster/profile analysis with the three MBI subscales, have been proposed by several research groups [13, 15]. However, until now, intra-individual patterns of burnout symptoms have primarily been investigated in non-clinical samples such as students, athletes, healthy, and burned-out employees. Therefore, the current study aimed to investigate whether different burnout subtypes can also be identified in a more homogeneous group of clinically diagnosed burnout patients enrolled in an employee rehabilitation program in a psychosomatic clinic. Our results provide evidence that in clinical burnout patients three different burnout subtypes can be revealed: the *exhausted* subtype, the *exhausted/cynical* subtype, and the *burned-out* subtype, which might represent patients at different developmental stages in the burnout cycle. Furthermore, the *exhausted* subtype displayed the lowest depression level and the best recovery/resources-stress balance, while the *burned-out* subtype exhibited the most severe depression symptoms and a worse recovery/resources-stress balance than the *exhausted* subtype. Clearly, the current findings need to be replicated in future studies involving larger, ideally longitudinal data sets to investigate the efficacy of rehabilitation interventions in different burnout subtypes and to track the stability and change patterns between the burnout subtypes over time.

## Abbreviations

ANOVA: Analysis of variance
BDI: Beck depression inventory
BDI-SF: Beck depression inventory – short form
CIS: Checklist individual strength
DSM-5: Diagnostic and statistical manual of mental disorders
ICD-10: International statistical classification of diseases and related health problems
JD-R: Job demands-resources
MANOVA: Multivariate analysis of variance
MBI: Maslach burnout inventory
MBI-GS: Maslach burnout inventory – general survey
MINI: Mini-international neuropsychiatric interview
PHQ-9: Patient health questionnaire
RESTQ-Work: Recovery-stress-questionnaire for work
SCID-I: Structured clinical interview for Axis I DSM-IV disorders
SCL-90-A: Symptom checklist – anxiety subscale
SCL-90-D: Symptom checklist – depression subscale
